# The Treatment of the Sick in Norwich during the Seventeenth Century

**Published:** 1903-12-05

**Authors:** 


### The Treatment of the Sick in Norwich During the Seventeenth Century.

Mr. Charles Williams, F.R.C.S.E., Senior Surgeon to the Norfolk and Norwich Hospital, has recently published a very interesting account of the treatment of the sick poor in Norwich during the seventeenth century, a time when as yet there were no hospitals or dispensaries. His information is derived from the assembly books of the Court of Aldermen of the City of Norwich from 1600 to 1700. These books are now preserved in the muniment room at the castle, and they contain many details relative to such poor people as were afflicted with various diseases, and more especially with that of stone. This complaint appears to have been a well recognised and prevalent disorder, as indeed it is still, not only in Norfolk but in the whole of East Anglia. The disease had probably existed in the district for hundreds of years, because the site of a large Roman cemetery containing sepulchral urns and other funeral vessels was discovered in 1828 at Litlington, Cambridgeshire. Within the bony pelvis of a skeleton was found a large oxalate of lime calculus which is now in the museum at Cambridge, and is the earliest known instance of a human calculus.

At Norwich it was the custom during the seventeenth century for such persons as suffered from that complaint to present themselves before the Mayor and Court of Aldermen with a view to solicit their help in procuring surgical assistance, as well as obtaining some pecuniary aid to support them in their convalescence. A certain routine was necessary to secure the services of an operator, and to ensure his being remunerated for his operation and the subsequent treatment of the case; the poor, in those times, were quite unable to pay the expenses incidental to a large operation such as that which was necessary for the removal of stone.

The principal mode of procedure was for the parent of the afflicted child or the patient himself, if he were an adult, to appear before the Mayor and Court of Aldermen at the Guildhall, and state the circumstances of the case to them. If the Court was satisfied with the information it received, it was then resolved that the aldermen of the ward in which the sufferer lived should be directed to have an interview with the Lord Bishop and place the facts of the case before him, and should then request him to direct the clergyman of the parish to which the sufferer belonged to make a collection on "the next and following Lord's daies" for the benefit of the person. Sometimes the aldermen themselves were ordered to make the collection, and occasionally the church-wardens and overseers were directed to make a house-to-house visitation. In some instances, several parishes combined to make up the required sum of money. Sometimes several Sundays were necessary to do so. On one occasion the friends of the patient were permitted to make a collection; and at another time the parents of the child were granted a similar permission. The money thus collected was generally handed over to the Mayor and Aldermen in some instances by the Swordbearer, but more often by the Alderman in whose hands the matter had been placed by the Court. A resolution was then passed giving directions as to the manner in which the sum of money which had been collected was to be distributed; how much the operator was to have, and how much was to be given to the parents and friends. The following are examples:—

"23 September, 1640.

"Ordered that £3 10s. be paid for cutting of John Terry, a poor child of St. George's parish, of the stone, and the doctor hath undertaken to do the cure for £3: 30s. to be paid in hand and 30s. more to be paid him when the child is cured, and 10s. to be given to the poor child's mother to be laid out in necessaries for him."

"11 February, 1670.

"It is ordered that papers be sent to the ministers of St. Peter of Mancroft, St. Andrew, St. Laurence, St. George of Tombland, and St. Stephen, to exhort them and their parishioners to contribute towards the charge of cutting the son of Wm. Carr of the stone and also towards the charge of the cure of Robert Horsfield of the same disease, which collection is to be made upon Sunday se'nnight, and the money to be brought to the Court.

"The Aldermen of the Ward of West Wymer are desired to treat with Mr. Gutteridge, the chyrurgeon, about the cutting of William Carr, his son."

"3 August, 1670.

"Mr. Swordbearer brought into the Court what money was collected for the cutting of the two boys of the stone, viz., in the parish of St. Laurence, 25s. 5d.; St. Stephen, 22s. 1½d.; St. Peter, Mancroft, 52s. 11d.; St. George, Tombland, 23s. 8d.; St. Andrew, 35s. 9½d., in all seven pounds 19 shillings and ten pence three farthings."

This admirable custom came to an end, says Mr. Williams, in 1711, when a Court of Guardians was incorporated by Act of Parliament. The principal objects of the Act were to order the erection of a workhouse in Norwich and to regulate the maintenance and care of the poor as well as to make provision for the sick.

